# Evidence for a spin acoustic surface plasmon from inelastic atom scattering

**DOI:** 10.1038/s41598-021-81018-9

**Published:** 2021-01-15

**Authors:** G. Benedek, M. Bernasconi, D. Campi, I. V. Silkin, I. P. Chernov, V. M. Silkin, E. V. Chulkov, P. M. Echenique, J. P. Toennies, G. Anemone, A. Al Taleb, R. Miranda, D. Farías

**Affiliations:** 1 Dipartimento di Scienza dei Materiali, Universitá di Milano-Bicocca, Via R. Cozzi 55, 20125, Milan Italy; 2 Donostia International Physics Center (DIPC), 20018 San Sebastián/Donostia, Basque Country Spain; 3 École Polytechnique Fédérale de Lausanne, 1015 Lausanne, Switzerland; 4 Tomsk State University, 634050 Tomsk, Russia; 5 Engineering School of Nuclear Technology, Tomsk Polytechnic University, 634050 Tomsk, Russia; 6 Departamento de Polímeros y Materiales Avanzados: Física, Química y Tecnología, Facultad de Ciencias Químicas, Universidad del País Vasco UPV/EHU, 20080 San Sebastián/Donostia, Basque Country Spain; 7 IKERBASQUE, Basque Foundation for Science, 48013 Bilbao, Basque Country Spain; 8 Centro de Fisica de Materiales, Centro Mixto CSIC-UPV/EHU, 20018 San Sebastian/Donostia, Basque Country Spain; 9 St. Petersburg State University, 198504 St. Petersburg, Russia; 10 Max-Planck-Institut für Dynamik und Selbstorganisation, Bunsenstraße 10, 37073 Göttingen, Germany; 11 Departamento de Física de la Materia Condensada, Universidad Autónoma de Madrid, 28049 Madrid, Spain; 12 Instituto Madrileño de Estudios Avanzados en Nanociencia (IMDEA-Nanociencia), 28049 Madrid, Spain; 13 Instituto “Nicolás Cabrera”, Universidad Autónoma de Madrid, 28049 Madrid, Spain; 14 Condensed Matter Physics Center (IFIMAC), Universidad Autónoma de Madrid, 28049 Madrid, Spain

**Keywords:** Physics, Condensed-matter physics, Electronic properties and materials

## Abstract

Closed-shell atoms scattered from a metal surface exchange energy and momentum with surface phonons mostly via the interposed surface valence electrons, i.e., via the creation of virtual electron-hole pairs. The latter can then decay into surface phonons via electron-phonon interaction, as well as into acoustic surface plasmons (ASPs). While the first channel is the basis of the current inelastic atom scattering (IAS) surface-phonon spectroscopy, no attempt to observe ASPs with IAS has been made so far. In this study we provide evidence of ASP in Ni(111) with both Ne atom scattering and He atom scattering. While the former measurements confirm and extend so far unexplained data, the latter illustrate the coupling of ASP with phonons inside the surface-projected phonon continuum, leading to a substantial reduction of the ASP velocity and possibly to avoided crossing with the optical surface phonon branches. The analysis is substantiated by a self-consistent calculation of the surface response function to atom collisions and of the first-principle surface-phonon dynamics of Ni(111). It is shown that in Ni(111) ASP originate from the majority-spin Shockley surface state and are therefore collective oscillation of surface electrons with the same spin, i.e. it represents a new kind of collective quasiparticle: a Spin Acoustic Surface Plasmon (SASP).

The dynamical properties of solids in the low-energy range (0–100 meV) are dominated by lattice vibrations (phonons). The interaction of phonons with the elementary excitations of the electronic degrees of freedom falling in the same energy range, such as Fermi-level electron-hole transitions in metals^1^, magnons in magnetic materials^2,3^ and excitons^4^ in narrow-gap semiconductors, is a very active research area in solid state physics.

More than six decades ago David Pines suggested that in a system with two types of electronic carriers with different Fermi velocities^5,6^ a low-energy collective electronic excitation with a sound-like dependence of energy on momentum—called acoustic plasmon—can be realized. Possible systems are, e.g., semiconductor surfaces with an inversion layer and quantum-well minibands at the Fermi level^7,8^. However, never such a mode was observed in metals, while the first experimental evidence of an acoustic surface plasmon (ASP) on a metal surface, Be(0001), was obtained with high-resolution electron-energy loss spectroscopy (HREELS)^9^. This was following Silkin et al. theoretical predictions of ASP associated to a Fermi level surface state band coupled to the bulk three-dimensional (3D) electron gas^10^. Similar observations have then been reported in Cu(111)^11^ and Au(111)^12,13^ and predicted to exist in many other systems^10^.

The energy range in the ASP probed up to now with HREELS is above $\approx$ 100 meV. However, a sound-like dispersion of the ASP means that its energy goes to zero at vanishing momentum. Hence, one can expect that at sufficiently low energies, the ASP dispersion enters into the energy domain of lattice vibrations, eventually resulting in a coupled dynamics of ASP with optical surface phonons. A key feature of systems supporting an ASP is the presence at the Fermi level of a shallow surface band with a binding energy in the range of a hundreds meV or less. This condition also holds for the Ni(111) surface for the majority-spin surface band, the minority-spin surface band being unoccupied^14,15^. In this case, a Spin Acoustic Surface Plasmon (SASP) is expected.

In this work evidence is given that the dispersion curves of THz SASP can be measured with inelastic atom scattering (IAS), together with those of surface phonons and possibly the SASP-phonon coupling within the phonon spectral region. This ability of IAS from conducting surfaces, enabling a new high-resolution spectroscopy of surface collective electron excitations in the low-energy domain hardly accessible to other surface probes, relies on the peculiar mechanism of exchanging energy and parallel momentum with the solid surface.

The scattering of neutral atoms at thermal energies from a metal surface occurs at no less than 0.3 nm above the first atomic plane. Due to the short-range nature of the surface-atom repulsive potential $V(\mathbf{r})$, approximately proportional to the surface electron density $n(\mathbf{r})$^16–19^, an incident neutral atom acts as a mechanical probe of the surface free-electron gas. This allows for surface-phonon spectroscopy, whereby all energy and momentum exchanges between probe and metal atoms occur basically via the phonon-induced modulation of $n(\mathbf{r})$, i.e., via the electron–phonon (e–p) interaction^18^. In this way, IAS can detect phonons within the range of e–p interaction, which can extend several layers beneath the surface (quantum sonar effect (QSE))^19^. The proportionality between $V(\mathbf{r})$ and $n(\mathbf{r})$ allows to express the IAS intensities as proportional to the Fourier transform of the time-dependent density-density autocorrelation function. The time dependent modulation of the surface change density $n(\mathbf{r})$ can be induced by phonons via electron–phonon interaction as well as by the collective excitations of the surface electron gas.

## Observation of a spin acoustic surface plasmon with Ne atom scattering

Ni(111) has actually been the very first metal surface investigated with IAS time-of-flight (TOF) spectroscopy from a metal surface. In these measurements, performed by Feuerbacher and Willis in 1981 with Ne-atom scattering (NeAS)^20^, evidence was obtained, besides the observation of the Rayleigh wave (RW) dispersion curve up to 2/3 of the surface Brillouin zone (SBZ) in the $[11\overline{2}]$ direction, of additional excitations at very small wavevectors and energies extending beyond the maximum of the phonon spectrum. These authors underlined the basic difference between IAS from insulator and metal surfaces, the latter involving surface conduction electrons in all energy and momentum exchanges with the solid, whereas IAS from insulator surfaces was well described by simple two-body interatomic potentials^21^. However, a possible association of the additional branch to single-particle electron–hole (e–h) excitations was, at about the same time, proved theoretically to be very unlikely due to the very weak cross-section, well below the sensitivity of conventional IAS spectroscopy^21–24^. Only in the spectral region where e–h excitations are strongly hybridized with surface phonons the corresponding sharp non-adiabatic anomalies could be observed with He-atom scattering (HAS)^25,26^ and explained in terms of single e–h surface excitations^27^. In the latter paper it was however anticipated that also low-energy surface collective electron excitations like two-dimensional (2D) plasmons and ASP would be within present IAS sensitivity.

The experimental proof of the existence of SASP mentioned in the introduction suggested that the unexplained branch observed with NeAS could be ascribed to these excitations. To prove this assignment, new NeAS and HAS studies of the Ni(111) surface together with calculations of the self-consitent surface-electron response function and of the first-principles surface phonon dispersion curves have been undertaken in the present work. This allows for a clear assignment of the inelastic features observed with both atom probes within the continuum of the surface-projected phonon to SASP. A density-functional perturbation theory (DFPT) calculation of the Ni(111) surface phonon dispersion curves, besides reproducing the experimental surface phonon branches, permits to assess the effects of SASP-phonon hybridization.

**Figure 1:**
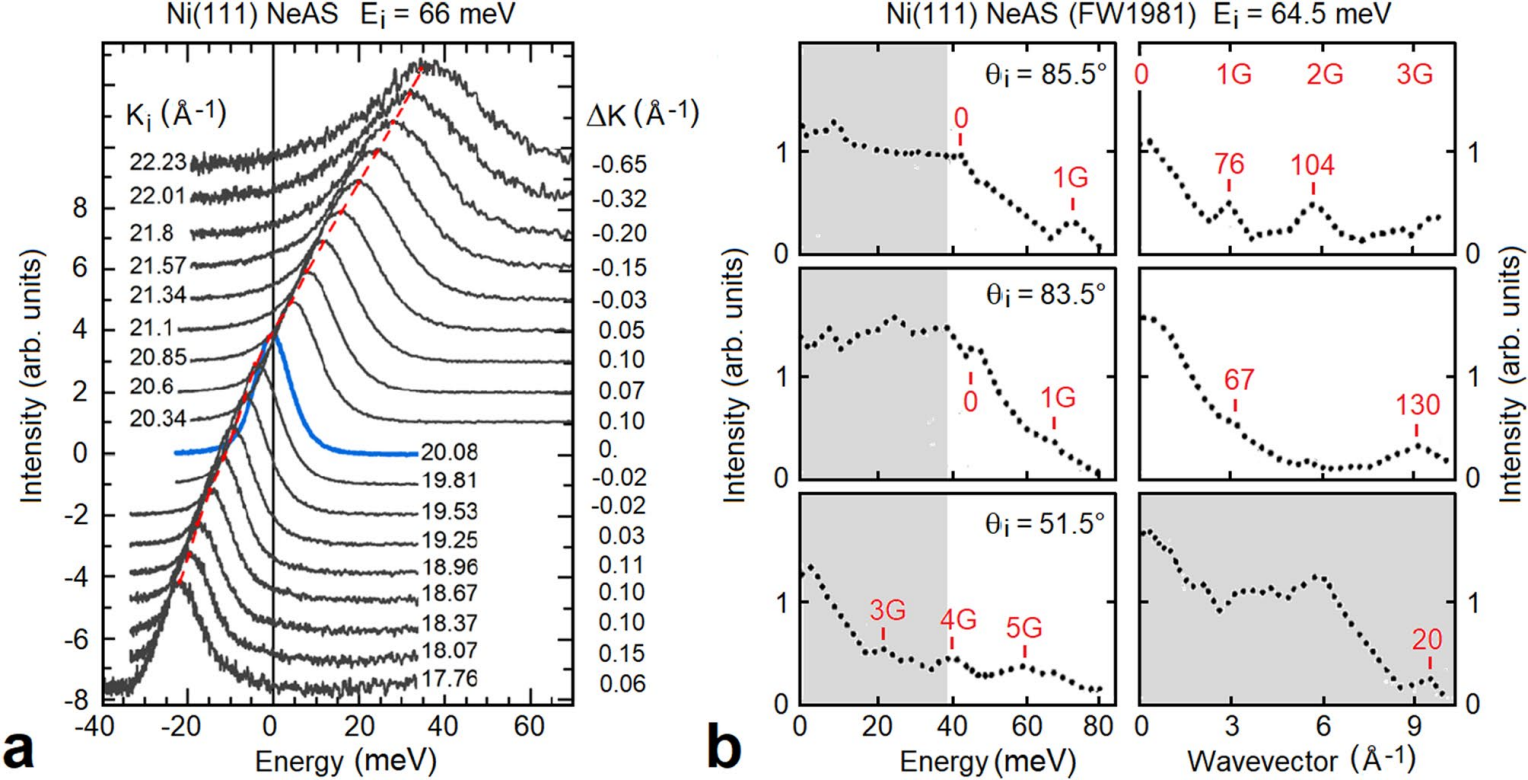
(**a**) NeAS time-of-flight (TOF) spectra, measured with an incident energy of 66 meV along the $\overline{\Gamma }\overline{\mathrm{M}}$ direction of the Ni(111) surface at a temperature of 400 K, plotted as a function of the energy transfer for different parallel incident wavevectors $K_i$ and a constant scattering angle of $105.4^{{\circ }}$. The incident angles vary at intervals of $1^{{\circ }}$ from $52.7^{\mathrm{o}}+9^{{\circ }}$ ($K_i = 22.23\,\AA ^{-1}$) to $52.7^{\mathrm{o}}-8^{{\circ }}$ ($K_i = 17.76\,\AA ^{-1}$) (specular condition corresponds to the blue curve). The parallel wavevector transfer $\Delta K$ corresponding to the maxima (broken red curve) of the inelastic peaks, show that the corresponding excitations occur at comparatively small wavevectors, compatible with the assignement to SASP. (**b**) Early inelastic NeAS spectra from Ni(111) in the $\overline{\Gamma }\overline{\mathrm{M}}$ direction by B. Feuerbacher and Willis^20^ as privately communicated to Bortolani and reproduced in Ref.^29^ in a preliminary theoretical study. The spectra are plotted vs. energy transfer (left) and wavevector transfer (right), for an incident energy of 65.4 meV, a fixed scattering angle of $135^{{\circ }}$ and the surface at room temperature (grey areas: bulk phonon region). The features assigned to SASP fall at about integer multiples of the reciprocal-lattice *G* vector (marked in left panel), also with energies (marked in right panel) well outside the phonon spectrum. The higher SASP energies can be observed on the annihilation side where noise is smaller; errors are estimated to be $\pm \,0.1\,\AA ^{-1}$ for wavevectors at 100 meV and $\pm \,2\,\hbox {meV}$ for energy.

A series of inelastic NeAS TOF spectra has been measured with an incident energy of 66 meV along the $\overline{\Gamma }\overline{\mathrm{M}}$ direction of the Ni(111) surface at a temperature of 400 K. The angle between the incident and scattered beams—the source-to-detector angle in planar geometry—is fixed at a total angle $\theta _{SD}=\theta _i+\theta _f=105.4^{{\circ }}$, while the incident angle varies by steps of $1^{{\circ }}$ from $52.7^{\mathrm{o}}+9^{{\circ }}$ (incident parallel wavevector $K_i = 22.23\,\AA ^{-1}$) to $52.7^{{\circ }}-8^{{}}$ ($K_i = 17.76\,\AA ^{-1}$). Hereafter, upper (lower)-case symbols shall be used for 2D (3D) vectors.

The resulting TOF spectra are plotted as functions of the energy transfer in Fig. 1a for the corresponding values of $K_i$. The parallel wavevector transfer $\Delta K$ corresponding to the maxima (broken red curve) of the inelastic peaks, are indicated aside the r.h. ordinate scale, and show that the corresponding excitations occur at comparatively small wavevectors. The attribution of these peaks to multi-phonon processes meets the following difficulty: in the limit of a Debye–Waller exponent $2 W>> 1$, as is the case in these experiments with a large incident energy and a large surface temperature, the full width at half maximum (FWHM) of the multi-phonon peak can be approximated by $(8\ln {2} \epsilon _{max}\epsilon _0)^{1/2}$, where $\epsilon _{max}$ is the peak maximum energy and $\epsilon _0$ is an average phonon energy^28^, and this, with, e.g., $\epsilon _{max} \sim \epsilon _0 \sim 20$ meV, gives FWHM = 47 meV, which is clearly much larger than observed. Indeed, as appears from the following analysis, the observed sequence of peaks can be safely attributed to SASP, with possible avoided crossings with surface optical phonon modes.

These new NeAS data from Ni(111) are here integrated by an analysis of the extended set of NeAS data (Fig. 1b), privately communicated by Feuerbacher to V. Bortolani and reproduced in their theoretical study^29^. This set actually shows a few additional data points at wavevector transfers close to *G*-vector multiples and energies up to 4 times the maximum phonon energy $\epsilon _M \approx$ 38 meV (Fig. 1b). The latter fact should exclude the possible association of the additional peaks to multiphonon processes^30^. The multiphonon background is clearly present beneath the one-phonon spectral region but does not seem to extend beyond $\pm 2\epsilon _M$ (cfr. Fig. 1b, left panels).

**Figure 2:**
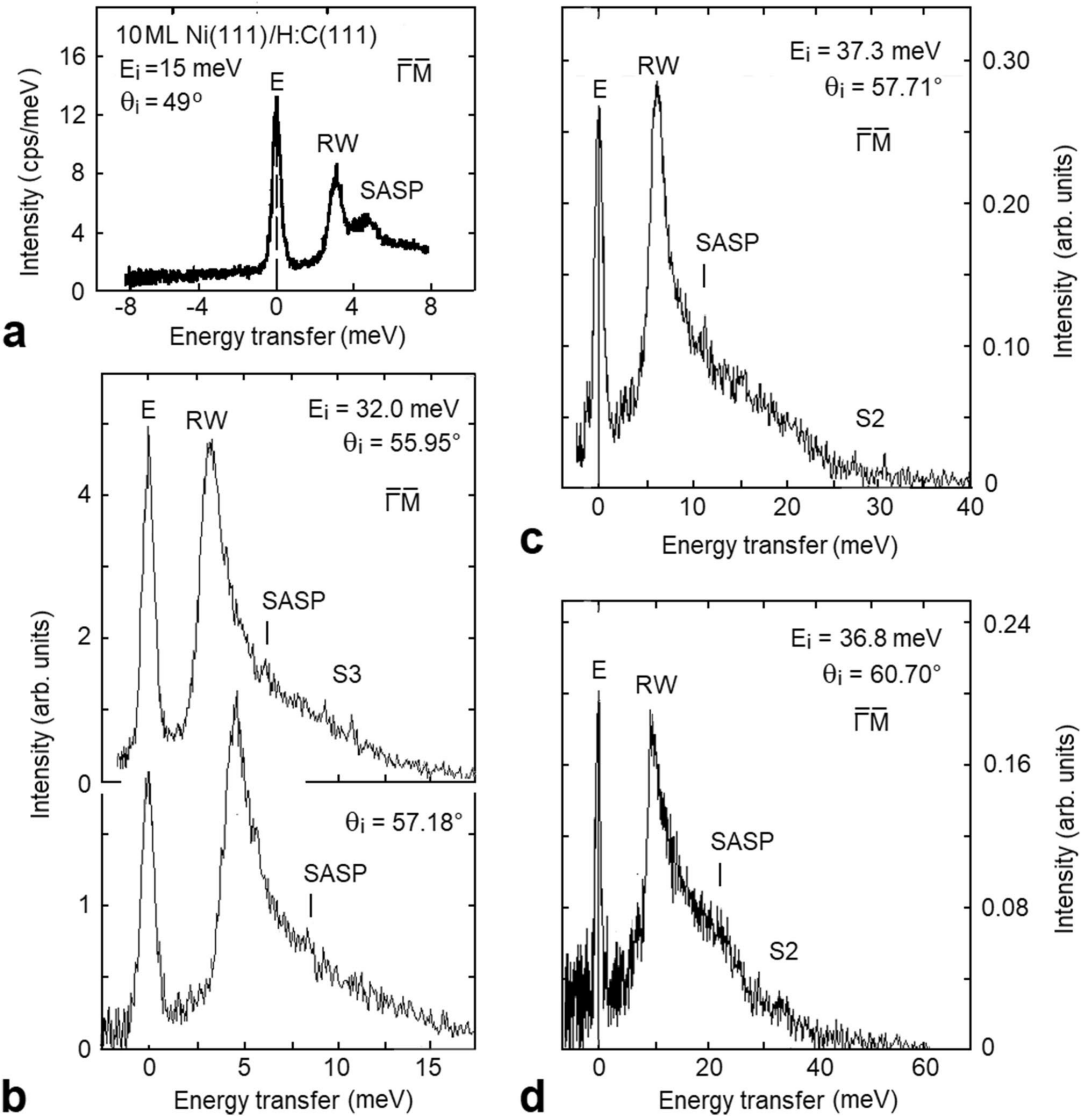
Selected HAS TOF spectra converted into energy transfer scale: (**a**) for a 10 ML film of Ni(111) grown on C(111) (from Ref.^31^) and (**b**–**d**) for a clean Ni(111) surface (present work). The SASP peak is more intense for the 10 ML film due to quantum-well confinement. Besides the Rayleigh wave (RW), narrow features corresponding to the SASP are clearly visible in the spectra recorded from Ni(111). The rather broad SASP feature seen at 21 meV reflects the avoided crossing with the S4 phonon (see Fig. 3a). Other smaller features, falling where the optical phonon modes S2 and S3 are expected, are visible above the background noise.

On the other hand, the observation of inelastic Umklapp processes with various G vectors is somewhat surprising in NeAS, since diffraction studies from metal surfaces normally do not reveal any appreciable surface corrugation. Actually the excitation of phonons by atom collisions on metal surfaces occurs through the creation of a virtual e-h pair, eventually recombining into a phonon^28^. The same holds for the creation of a SASP, and the matrix element converting an e-h pair into a SASP is governed by a potential reflecting lattice periodicity, and therefore allowing for *Umklapp* processes. This supports the assignment of the additional and long mysterious steep dispersion curve observed in Ni(111) to SASP. Also subsequent HAS data from a 10 ML Ni(111) film grown on diamond^31^ showed additional modes (see, e.g., the TOF spectrum in Fig. 2a) forming a very steep dispersion curve, which was tentatively ascribed to the diamond substrate. However, the steepness of that additional branch, much larger than that of the diamond RW^32^ provides a further argument in favour of Ni(111) SASP. It is interesting to note that in similar NeAS measurements on Ir(111)^33^, where the surface electronic structure at the Fermi level does not support any ASP, the observed peak dispersion and FWHM can actually be fairly well assigned to multiphonon processes.

## Surface phonons and SASP of Ni(111) from He atom scattering

A comparison between two of the present TOF spectra (Fig. 2b) and a HAS spectrum for the 10ML-Ni(111) film measured by Braun et al.^31^, reproduced in Fig. 2a, (all converted to the energy-transfer scale) shows, aside the diffuse elastic peak (E) and the dominant RW peak, smaller features at wavevector $Q \sim$ 0, ascribed to SASPs. Note that the SASP localization within the 10 ML quantum well can justify the comparably larger SASP intensity in the film than in the semi-infinite crystal. Panels (c, d) of Fig. 2 show other examples of SASP features seen at higher energies, but still within the phonon spectrum.

**Figure 3:**
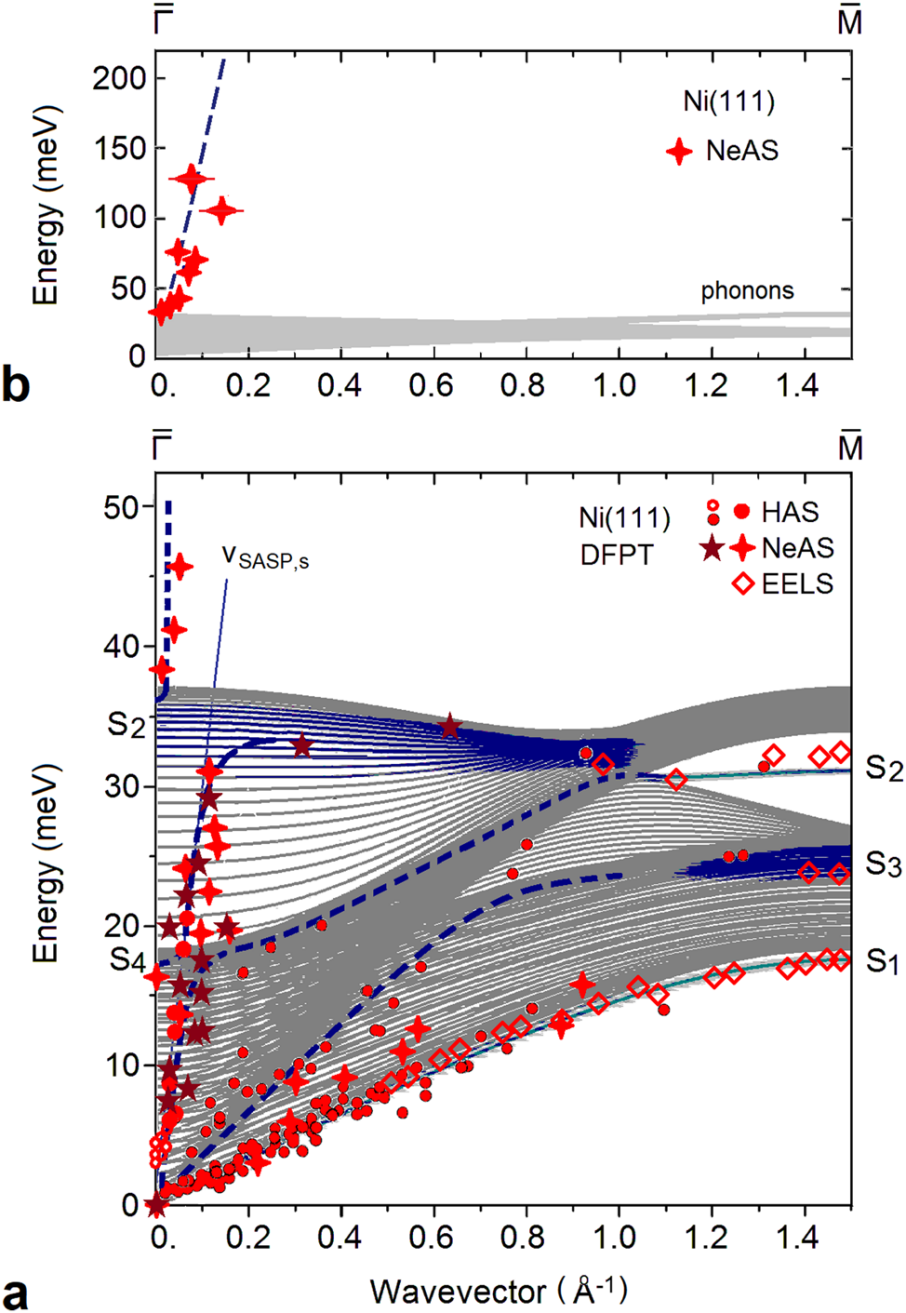
(**a**) The set of inelastic scattering data from HAS (full and empty circles), NeAS (five-stars: present; crosses: Refs.^20^ and^34^) and EELS (empty lozenges^35^) in the low energy region, superimposed to the DFPT calculation of Ni(111) dispersion curves (full lines: surface phonons with $\hbox {S}_1$ the Rayleigh wave, $\hbox {S}_2$ the optical Wallis mode and $\hbox {S}_4$ the sagittal Lucas mode; blue area: surface phonon resonances, with $\hbox {S}_3$ the longitudinal acoustic surface resonance; broken lines are eye guidelines showing the possible SASP-surface phonon avoided crossings and the SASP velocity $\mathrm{v}_{\mathrm{SASP,s}}$ in the phonon region). (**b**) NeAS data points above the phonon maximum from Refs.^20,29^, with the broken line indicating the Fermi velocity of the Ni(111) majority-spin surface state.

The full sets of present and past HAS (full and open circles) and NeAS data (five-stars: present; crosses: Refs.^20,34^) measured along [11$\bar{2}$] are plotted in Fig. 3 after folding into the positive quadrant of the first SBZ, together with the available HREELS data (lozenges) by Menezes et al.^35^. In Fig. 3a the experimental data are superimposed for comparison to ab initio calculation based on DFPT^36^. The surface dynamics of Ni(111) has been extensively investigated theoretically on the basis of force-constant models^29,35,37–46^ of effective-medium theory^47^, of a first-principle Green’s function method^48^, and of molecular dynamics with the Finnis–Sinclair potential^49^. To our knowledge, the present calculation displayed in Fig. 3 is the first one performed with DFPT. The good agreement with the calculation permits a clear assignment of the observed phonons to the RW, the anomalous resonance S3, the sagittal Lucas resonance S4 and the optical Wallis S2 branches^50^. In particular, the DFPT calculation reproduces very well the EELS data^35^ at the zone-boundary for the RW (S1), the S3 resonance and the optical gap mode S2.

### SASP-phonon coupling

The plot in Fig. 3a, b of the full sets of HAS and NeAS data, whether assigned to phonons or SASP, allows to appreciate the effects of the SASP-phonon coupling. The broken-line eye-guides interpolating the data points suggest, in Fig. 3a,possible avoided crossings between the steep linear dispersion of SASP and the phonon branches, and in Fig. 3b on a compressed energy scale, the continuation of the SASP branch above the phonon spectrum (shown as a grey area).

While at high energy the slope of the SASP branch is close to the calculated Fermi velocity of the surface majority-spin electronic band (broken line in Fig. 3b), the SASP branch running inside the phonon spectrum (Fig. 3a) exhibits a considerable slower velocity due to the strong e-p coupling which yields a larger carrier effective mass. In other words, the collective carrier oscillations drag the atoms due to the coincidence of the SASP energy and momentum with those of some bulk phonon of the spectrum. The same coupling leads to the avoided crossing with the surface-localized phonons, and a gradual change of an SASP into a phonon and viceversa. These mixed modes have been termed plasmarons in the original works on semiconductor quantum wells^8^. A similar hybridization is expected to occur for SASP dispersion curves and the surface optical phonon branches of other metal surfaces (notably with the shear-vertical (S2) and longitudinal Lucas (S4) optical modes).

## Theory of atom scattering from spin-acoustic surface plasmons

### Distorted-wave Born approximation

The way an incident closed-shell atoms can exchange energy and momentum with a metal surface can be understood within the Esbjerg–Nørskov (EN) approximation for the atom-surface potential^16^. In this approximation only the steep repulsive part $V(\mathbf{r},t)$ of the atom-surface potential energy is considered and written in the form

1 $$\begin{aligned} V(\mathbf{r},t) \cong A_n n(\mathbf{r},t), \end{aligned}$$

where $n(\mathbf{r},t)$ is the surface electron density and $A_n = 364$ eV/a.u. is the Esbjerg–Nørskov constant as currently used for He atoms on metal surfaces^51^. A different value of $A_n$ is expected for Ne, due to its larger attractive dispersion forces and larger size with respect to He. If one adopts the simple EN potential, these contributions are accounted for by an effective EN constant, which is therefore dependent on the probe atom. There is however a large discrepancy between the theoretical value of $A_n$ obtained for Ne by Puska et al. (which IAS quite larger than that of He)^52^, and the value, much smaller than that of He, derived by Baumberger et al. from experiments^53^. This discrepancy may be attributed to the difficulty of earlier density functional methods to account for dispersion forces. It is noted, however, that it concerns the constant prefactor in Eq. (3) and is therefore irrelevant for the present discussion. The time dependence of $n(\mathbf{r},t)$ due to the surface elementary excitations determines the energy exchange, as well as the dynamical space modulation determines the exchange of parallel momentum with the surface. The differential reflection coefficient in the distorted-wave Born approximation (DWBA), for an IAS casting the atom of incident energy $E_i$ and normal wavevector $k_{iz}$ into the final solid angle $\Omega _f$ with final energy $E_f$ and wavevector $k_f$, is proportional to the Fourier transform of the potential-potential autocorrelation function^28^:

2 $$\begin{aligned} \frac{\mathrm{d}^2\mathfrak {R}}{\mathrm{d}E_f \mathrm{d}\Omega _f} \propto \frac{k_f}{|k_{iz}|} \int _{-\infty }^{+\infty } \mathrm{d}t e^{-i(E_f-E_i)t/\hbar } \langle V_{fi}(0) V_{fi}^+(t)\rangle , \end{aligned}$$

where $V_{fi}(t)$ is the time-dependent matrix element of the potential between the final and the incident atom wavefunctions as distorted by the static surface potential. Let $\hbar \omega =E_i-E_f$ and $\mathbf{Q}=\mathbf{K}_i-\mathbf{K}_f-\mathbf{G}$ be the energy and parallel wavevector of the surface elementary excitation created in the IAS process, where $\mathbf{K}_i$ and $\mathbf{K}_f$ are the parallel components of the incident and final atom wavevectors, respectively, and $\mathbf{G}$ is a surface reciprocal lattice vector. In the EN approximation, Eq. (1), one actually needs the autocorrelation function of the charge density matrix elements $n_{fi}(t)$. For a static flat surface, as generally found for close-packed metal surfaces such as Ni(111) and for small incident energies $E_i$, the static surface charge density only depends on the coordinate *z* normal to the surface: $n_0(\mathbf{r})=n_0(z_t)e^{-\kappa z}$, where the He-surface classical turning point $z_t = z_t(E_i)$ is taken as the origin of *z*, and $\kappa$ is the repulsive parameter. The matrix element $A_n n_{0fi}(\mathbf{r})$ between final and incident distorted waves of the corresponding static exponential potential can be calculated analytically in the DWBA^54^, which allows to express the IAS differential reflection coefficient as

3 $$\begin{aligned} \frac{\mathrm{d}^2\mathfrak {R}}{\mathrm{d}E_f \mathrm{d}\Omega _f} \propto \frac{k_f}{|k_{iz}|} S_{fi}^2 A_n^2 g(\mathbf{Q},\omega ), \end{aligned}$$

where

4 $$\begin{aligned} g(\mathbf{Q},\omega ) = \frac{1}{2\pi } \int _{-\infty }^{+\infty } \mathrm{d}t e^{i\omega t} \langle n^{\dagger }(\mathbf{Q},t) n(\mathbf{Q},0)\rangle \end{aligned}$$

is the Fourier transform of the dynamic density–density autocorrelation function, providing the response function of the surface electron density, and

5 $$\begin{aligned} S_{fi}= \frac{p_f^2-p_i^2}{\sinh ^2{p_f} - \sinh ^2{p_i}} \left( \frac{\sinh {2p_f} \sinh {2p_i}}{4p_fp_i} \right) ^{1/2}, \, p_f\equiv \pi \,|k_{fz}|/\kappa , \, p_i\equiv \pi \,|k_{iz}|/\kappa \,, \end{aligned}$$

is the Jackson-Mott kinematic factor. For a hard-wall potential ($\kappa \rightarrow \infty$) $S_{fi} = 1$.

**Figure 4:**
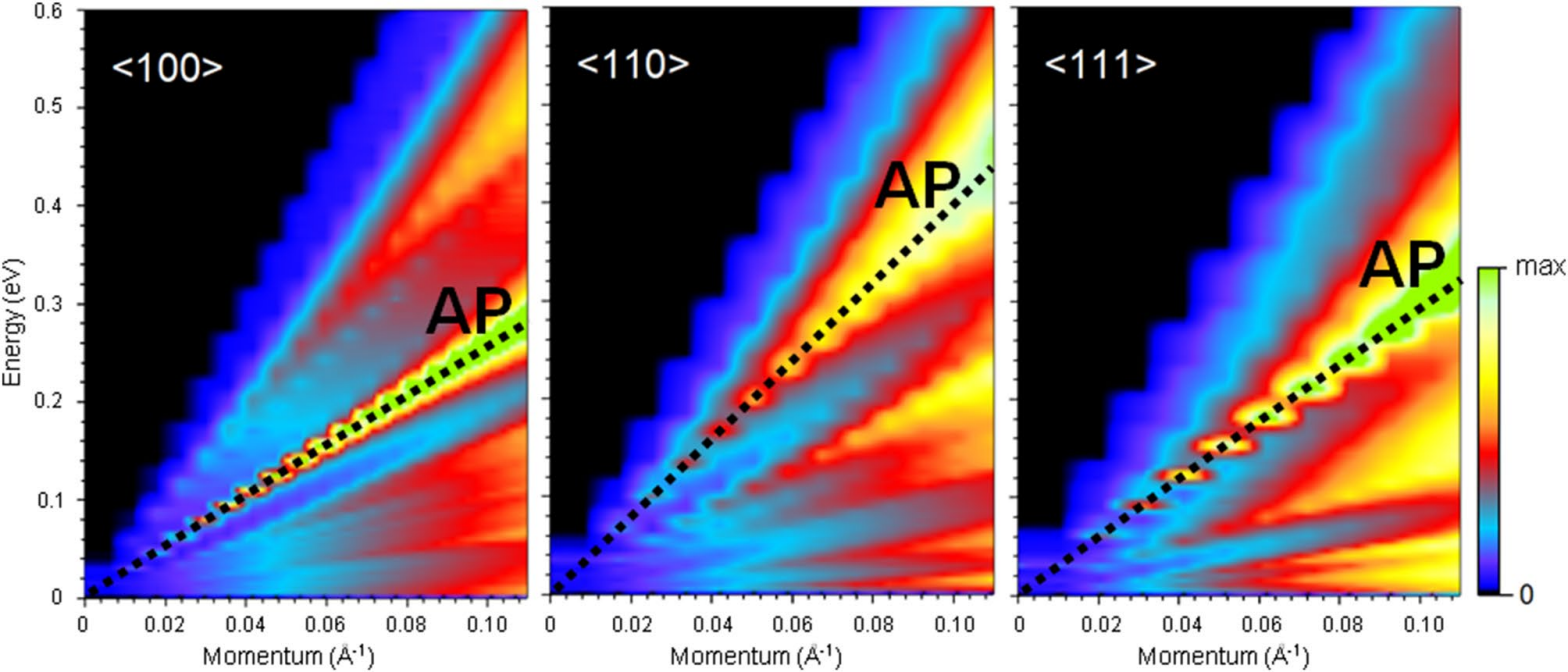
Normalized loss function, $N(\mathbf{Q},\omega )$, of bulk Ni in three symmetry directions, ${<}100{>}, {<}110{>}$, and ${<}111{>}$, of the face-centered-cubic lattice. Dotted lines highlight the bulk acoustic plasmon (AP) dispersion.

As far as it concerns the use of the above equations for NeAS, it is noted that Jackson–Mott theory provides an analytical form of the scattering matrix element for a negligible surface corrugation and an exponential form like the EN potential. These approximations allow for the factorization of Eq. (3), thus separating what is relevant in the present analysis, the density autocorrelation function. Since also Ne diffraction peaks for fcc(111) metal surfaces are found to be much smaller than the specular peak, the assumption of a flat surface is reasonable also for NeAS, so that the EN exponential potential has been adopted in the past also for NeAS with the appropriate constant^53^. Although the J–M kinematic factor does not account for some of the features mentioned above of real surface potentials, like dispersion forces and corrugation effects, the approximations made in deriving Eq. (3) should provide a sufficient basis for a first theoretical analysis of the SASP dispersion relations. As regards the kinematic factor, it is noted, however, that the hard-wall potential model (where $S_{fi} = 1$) is inappropriate for metal surfaces, especially for Ne, and therefore the prefactor $S_{fi}^{2}$ needs to be included due to its dependence on the initial and final momenta of the probe atom.

### Ni bulk and surface response functions

In this work, we explore two routes to determine a possible collective excitation in the electronic system responsible for the experimental observations. One, based on the first-principle calculations, consists in looking for the possibility of such low-energy excitations in the electronic system of bulk nickel. The second step consists in the evaluation of the surface response function of Ni(111) associated to the IAS. This calculation shows that the shallow majority-spin Shockley surface state occurring around the SBZ center supports the occurrence of a SASP branch.

**Figure 5:**
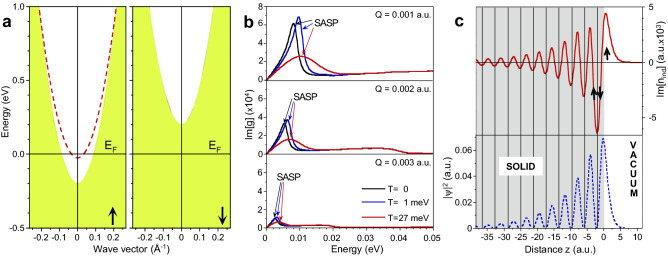
(**a**) Surface valence band structure of Ni(111) reproduced by a model potential. The majority-spin Shockley surface state dispersion is shown by red dashed line. The shaded regions show the projected bulk electronic state continua. Left (right) panel shows the majority- (minority-) spin part. (**b**) Surface loss function, $\hbox {Im}[g(Q,\omega )]$, calculated at $Q=0.001$, 0.002, and 0.003 a.u. Black, blue, and red curves show it for three values of electron temperature, 0, 1, and 27 meV, respectively. Peaks corresponding to the spin acoustic surface plasmon are marked as SASP. (**c**) Imaginary part of charge density corresponding to the acoustic surface plasmon at $Q=0.001$ a.u. and $\omega =2.6\,\hbox {meV}$ (red solid line). Blue dashed line shows charge density of the majority-spin Shockley surface state. Vertical solid lines indicate the atomic layer positions. Origin of the horizontal coordinate locates at a nominal crystal surface.

The calculated normalized bulk loss function $N(\mathbf{q},\omega ) \equiv L(\mathbf{q},\omega )/\omega$ at the momentum transfers $\mathbf{q}$ along three symmetry directions are displayed in Fig. 4. One can find several peaks with an acoustic dispersion in $N(\mathbf{q},\omega )$ in this momentum-energy region. Their existence can be explained by the presence of several energy bands crossing the Fermi level with different velocities. Detailed analysis showed that the prominent peaks, highlighted by dotted lines, can be considered as a signature of a bulk acoustic plasmon. Eventually, upon approaching the $\mathbf{q}=0$ limit its energy vanishes. However, its group velocities in all three directions are relatively large, substantially exceeding that of the mode interacting with phonons (Fig. 3) observed in the experiment.

In order to address possible electronic collective excitations at the surface of the solid probed by atom scattering one should evaluate the response function $g(\mathbf{Q},\omega )$, defined in Eq. (4), and here re-written as the Fourier transform of the electronic susceptibility:

6 $$\begin{aligned} g(\mathbf{Q},\omega ) = - 2\pi \int \mathrm{d}{} \mathbf{r} \mathrm{d}{} \mathbf{r}' \chi (\mathbf{r},\mathbf{r}',\omega ) e^{\mathrm{i}{} \mathbf{Q}(\mathbf{R}-\mathbf{R}')}. \end{aligned}$$

Its imaginary part, $\hbox {Im}[g(\mathbf{Q},\omega )]$, determines the differential cross section for the scattering with energy $\omega$ and in-plane momentum transfer $\mathbf{Q}$ and coincides with the surface loss function^55,56^ at $\mathbf{Q}=0$.

In the surface loss function, $\hbox {Im}[g(\mathbf{Q},\omega )]$, reported in Fig. 5b one can observe a peak labeled as SASP which we ascribe to the spin acoustic surface plasmon. We attribute its origin to the partly occupied majority-spin surface state, Fig. 5a. The Fermi velocity in this surface state band is significantly lower in comparison with that in the *sp* bulk energy bands. The resulting incomplete dynamical screening, involving slow carriers in the surface state band and the faster ones in the bulk electron system, is the origin of this mode^57^.

In Fig. 5b one can see that the variation in temperature *T* introduces notable modifications in the calculated surface loss function. First, with increasing *T* the SASP peak shifts to larger energies. Second, the broadening of the SASP peak increases as well. Nevertheless, even at room temperature, the SASP peak can be resolved in the calculated surface loss function.

In the upper panel of Fig. 5c we show the calculated imaginary part of the induced density, Im[*n*_ind_], corresponding to the SASP at $Q=0.001$ a.u. and $\omega$=2.6 meV. One can see, that the peak positions in Im[*n*_ind_] correspond to the charge density distribution observed in the majority-spin Shockley surface state reported in the bottom panel of Fig. 5c. As such, the positive parts in Im[*n*_ind_] of Fig. 5c have mostly majority-spin orientation. This is schematically shown by the upward arrow. On the contrary, the negative parts of Im[*n*_ind_] corresponding to screening by faster bulk electrons contain contributions from both the majority- and minority-spin electrons. Therefore, in a first approximation, this part in Im[*n*_ind_] can be considered as paramagnetic. Since, the total charge in Im[*n*_ind_] is almost zero due to the quadrupole nature of the SASP, the resulting spin of the oscillating charge related to the SASP should have excess of the majority-spin type. Moreover, since in the vacuum side the majority-spin surface state dominates, the oscillating charge related to the SASP should be mostly majority-spin resolved.

**Figure 6:**
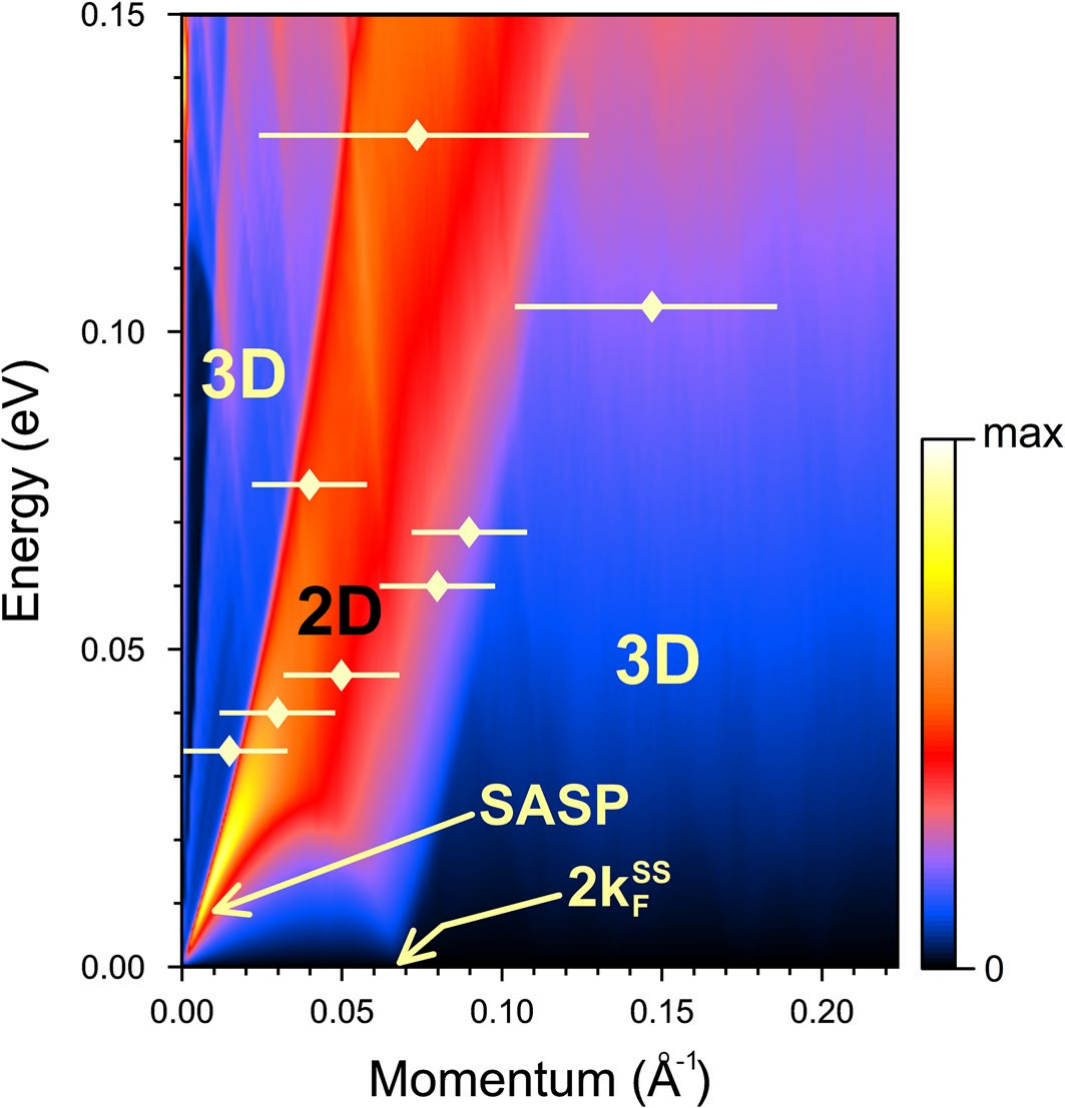
Calculated surface loss function, $\hbox {Im}[g(Q,\omega )]$, of the Ni(111) surface divided by *Q*. Region in the *Q*-$\omega$ plane dominated by single-particle intraband excitations in the majority-spin surface state is labeled as 2D. The regions where the electronic excitations within the bulk electronic system are available only are marked as 3D. A peak marked as SASP corresponds to the spin acoustic surface plasmon. The experimental data are shown by diamonds with horizontal lines representing the experimental uncertainty.

In Fig. 6 we report the two-dimensional plot of the calculated surface loss function as a function of *Q* and $\omega$. The area in $\hbox {Im}[g(Q,\omega )]$ denoted as 2D and corresponding mainly to intra-band excitations inside the majority-spin surface state clearly has larger amplitude in comparison to the regions where only excitations inside the bulk system are allowed. The Ne scattering data substantially fall into the 2D area of the electronic surface loss function. Due to the comparatively large experimental uncertainty it cannot be decided how far is the predicted SASP dispersion curve reproduced by experiment. On the other hand, the early conclusion by Schönhammer and Gunnarsson^21,22^ about the very small IAS cross-section of single e-h excitations, supports the assignment of the observed inelastic processes at small wavevector transfers and energy rapidly increasing well beyond the phonon maximum energy to electron collective excitations, notably to SASP. Thus, the change of slope from inside to outside the phonon spectrum, as well as the apparent gap between 30 and 35 meV associated to the avoided crossing between the SASP and the S2 phonon branch, can be attributed to e–p interaction. This is compatible with the comparatively large value of the mass-enhancement factor in nickel ($\lambda = 0.56$)^58^ derived from the temperature dependence of the HAS Debye–Waller exponent^59^.

## Conclusions

The analysis of inelastic atom scattering data from the (111) surface of nickel, based on calculations of the electronic response function and the surface phonon dispersion curves proves IAS as a valuable tool to investigate ASPs in the THz region, that is hardly accessible to other spectroscopies. The experimental findings were explained by a self-consistent calculation of the bulk and surface response functions and of the first-principle surface-phonon dynamics of Ni(111). We have shown that in Ni(111), the ASP originates from the majority-spin Shockley surface state and is therefore a collective oscillation of surface electrons with the same spin, i.e. it represents a new kind of collective quasiparticle: a spin acoustic surface plasmon (SASP). Present monochromatic atom scattering spectrometers, based on supersonic beams with a very high speed ratio, definitely attain a far better resolution than available in the early 80s, and are therefore suitable to investigate surface plasmon excitations in the THz frequency domain. $^3\hbox {He}$ spin-echo (3HeSE) spectrometry, in particular, with its unprecedented resolution in the neV range, can in principle measure the ASP dispersion in the GHz domain. Recent HAS experiments have provided the dispersion curves of other THz electronic collective excitations such as phasons and amplitons occurring in charge density wave systems^60,61^ and, with $^{3}$HeSE, in the long-period reconstructed $\hbox {Au}(111)23\times \sqrt{3}$ surface^62^. As shown in the case of $\hbox {Bi}_2\hbox {Se}_3$(111), bound-state resonance enhancement can be exploited in HAS experiments to amplify the response of low-energy electronic collective excitations^61^. On the other hand, fast neutral atom beams in grazing-incidence scattering experiments in the keV energy domain have also proved to have sufficient resolution to detect surface excitons in insulators^63^ and plasmon coherent excitations at metal surfaces^64^: nothing prevents in principle their use at smaller incident energy and higher monochromaticity for ASP at metal surfaces.

## Methods

### Experimental details

The present HAS measurements have been performed with a high-resolution HAS TOF spectrometer described in detail elsewhere^65^. A pulsed atom beam is produced by free jet expansion of the high pressure gas and a rotating disk chopper. The atoms scattered from the sample travel through three differential pumping stages along the 1.7-m-long drift tube before reaching the detector. The angle between the incident and scattered beams (the source-to-detector angle in planar geometry) is fixed at a total angle $\theta _{SD}=\theta _i+\theta _f=105.4^{{\circ }}$. The low intensity of the diffuse elastic peak indicates the high quality and cleanness of the crystal surface. The TOF spectra were integrated for typically 60–70 min at a surface temperature $T = 460$ K to avoid surface contamination with $\hbox {H}_2$. We discussed in more detail the TOF spectra and phonon modes of the Ni(111) single crystal in an earlier work^66^.

### DFPT calculations

The DFPT calculations were carried out using the Quantum-ESPRESSO package^67^. An ultrasoft pseudopotential^68^ with ten valence electrons and the Perdew–Burke–Ernzerhof (PBE) approximation^69^ for the exchange-correlation energy functional were used. The Kohn–Sham orbitals were expanded in plane waves up to an energy cutoff of 45 Ry while an 800 Ry cutoff was used for the charge density. A Gaussian smearing of 0.02 Ry was introduced in the occupation of states to deal with the metallic character of Ni while collinear spin-polarization was used to describe its ferromagnetic ground state. We optimized the bulk geometry by integrating the Brillouin zone (BZ) over a $12 \times 12 \times 12$ Monkhorst–Pack (MP) mesh^70^ obtaining an equilibrium lattice parameters of 3.518 Å. The surface phonon dispersion curves were first calculated for a 12-layer slab separated by its periodic replica by 25 Å of vacuum. The hexagonal SBZ was sampled over a $12 \times 12 \times 1$ MP grid. Atomic positions were relaxed until forces became lower than 0.1 mRy/bohr and phonons were computed on a $4 \times 4 \times 1$ grid. In order to obtain denser bands, thicker 38 layers slab was constructed through the insertion of a 26 layers bulk slab into the middle of the original 12-layer slab. The surface 6-layer slabs are sufficient to make the effect of the inserted bulk layers on the surface phonon negligible.

### Bulk and surface response functions calculations

A way to obtain information on the excitations in a bulk electron system is established in the framework of time-dependent density functional theory^71,72^. The well-defined peaks in the loss function $L(\mathbf{q},\omega ) \equiv -\mathrm{Im}[\epsilon ^{-1}_{\mathbf{g}{} \mathbf{g}}(\mathbf{q}',\omega )]$, directly probed in the energy-loss experiments, correspond to the plasmonic excitations. Here $\mathbf{q} \equiv \mathbf{g}+\mathbf{q}'$ with $\mathbf{g}$ being the bulk reciprocal lattice vectors. Vector $\mathbf{q}'$ is chosen to be inside the BZ. The inverse dielectric-function matrix $\epsilon ^{-1}_{\mathbf{g}{} \mathbf{g}}(\mathbf{q}',\omega )$ is related to the density-response function of interacting electrons $\chi$ as

7 $$\begin{aligned} \epsilon ^{-1}_{\mathbf{g}{} \mathbf{g}'}(\mathbf{q}',\omega ) = \delta _{\mathbf{g}{} \mathbf{g}'} \, + \,\chi _{\mathbf{g}{} \mathbf{g}'}(\mathbf{q}',\omega )V_{\mathbf{g}'}(\mathbf{q}'), \end{aligned}$$

where $V_{\mathbf{g}'}(\mathbf{q}')=4\pi /|\mathbf{q}'+\mathbf{g}'|^2$ is the Fourier transform of the bare Coulomb potential. $\chi _{\mathbf{g}\mathbf{g}'}(\mathbf{q}',\omega )$ is obtained from the matrix equation

8 $$\begin{aligned} \chi _{{\mathbf{g}}{} \mathbf{g}'}(\mathbf{q}',\omega ) = \chi ^o_{{\mathbf{g}}{\mathbf{g}}'}({\mathbf{q}}',\omega ) + \sum _{{\mathbf{g}}_1,{\mathbf{g}}_2} \chi ^o_{{\mathbf{g}}{} {\mathbf{g}}_1}({\mathbf{q}}',\omega )[V_{{\mathbf{g}}_1}\delta _{{\mathbf{g}}_1{\mathbf{g}}_2}+K^{\text{xc}}_{{\mathbf{g}}_1{\mathbf{g}}_2}({\mathbf{q}}',\omega )] \chi _{{\mathbf{g}}_2{\mathbf{g}}'}({\mathbf{q}}',\omega ). \end{aligned}$$

The kernel $K_{\mathrm{xc}}$ accounts for the exchange-correlation effects, which are set to zero within the random-phase approximation (RPA) employed here. In general, impact of these effects beyond the RPA in the dielectric properties of solids is small at small momentum transfers $\mathbf{q}$ which are of interest in this work. In the above equation, $\chi ^o(\mathbf{r},\mathbf{r}',\omega )$ is the response function of the non-interacting Kohn-Sham electrons, which in the reciprocal space reads

9 $$\begin{aligned} \chi ^o_{{\mathbf{g}}{} {\mathbf{g}}'}({\mathbf{q}}',\omega ) = \frac{1}{\Omega }\sum ^{\text{BZ}}_{{\mathbf{k}},s} \sum ^{\text{occ}}_{n} \sum ^{\text{unocc}}_{n'} \frac{f_{ns\mathbf{k}}-f_{n's\mathbf{k}+\mathbf{q}'}}{\varepsilon _{ns\mathbf{k}}-\varepsilon _{n's\mathbf{k}+\mathbf{q}'}+(\omega +\mathrm{i}\eta )} \langle {\psi _{ns\mathbf{k}}} |e^{-\mathrm{i}(\mathbf{q}'+\mathbf{g})\mathbf{r}}|\psi _{n's\mathbf{k}+\mathbf{q}'}\rangle \langle {\psi _{n's\mathbf{k}+\mathbf{q}'}} |e^{\mathrm{i}(\mathbf{q}'+\mathbf{g}')\mathbf{r}}|\psi _{ns\mathbf{k}}\rangle . \end{aligned}$$

Here $\Omega$ is the normalization volume, $f_{ns\mathbf{k}}$ is the Fermi occupation number, and summation over spin *s* is explicitly included. The sampling over $\mathbf{k}$ in the BZ was performed on a $300\times 300\times 300$ mesh. The one-particle energies $\varepsilon _{ns\mathbf{k}}$ and wave functions $\psi _{ns\mathbf{k}}$ of Ni were obtained in the electron density functional theory formalism implemented in the Vienna Ab-initio Simulation Package (VASP)^73–75^. Since we are interested in the plasmons determined by the intra-band transitions^5,57^, in Eq. (9) we include only such transitions. The corresponding matrix elements were set to unity. The occupied (occ) and unoccupied (unocc) states in the energy bands crossing the Fermi level were taken into account. In the Fourier expansion of $\chi ^o$, $\chi$, and $\epsilon ^{-1}$ we employed the $\mathbf{g}=0$ vector only, i.e. the local field effects were not considered, which in the low-$\mathbf{q}$ and low-$\omega$ domain is a good approximation. Other calculation details can be found, e.g. in Ref.^76^.

Nowadays to perform a full ab initio calculation of $g(\mathbf{Q},\omega )$^77^ for a Ni surface with a meV energy resolution using a sufficiently thick slab represents a formidable task. Therefore we employed significantly less time-demanding approach based on a model potential^78^ capturing essential features of the surface electronic structure of Ni(111). We constructed a spin-resolved potential for the Ni(111) surface following the recipe of Ref.^78^. In particular, the bulk energy gap positions in both spin channels and the *sp* Shockley surface state in the majority-spin case were taken into account. The resulting model band structure of the Ni(111) surface around the Fermi level obtained with this potential is presented in Fig. 5a. The data for the fitting of the model potential parameters were taken from the density-functional theory (DFT) calculations^15,79–81^. Since the presence of the minority-spin *sp* surface state in the Fermi level vicinity is not confirmed by the DFT calculation, it was not taken into account in the model potential construction.

Notice that there is a large spread in the energy positions of the Shockley surface states in different experiments. For instance, the absolute binding energies of the majority-spin surface state at the band bottom range from 225 meV in the scanning tunneling microscopy measurements^14^ to almost zero in the photoemission experiment^82^. It was pointed out in Ref. ^83^ that the energy position of this surface state is strongly temperature dependent. In turn, the Fermi velocity $v_F$ of the surface state changes as well. In consequence, the SASP dispersion, that is proportional^57^ to $v_F$, may be sensitive to the factors determining the surface state energy position. Our fitted Ni(111) electronic structure is close to that measured in the spin-resolved photoemission experiment carried out at room temperature^83^.

With this model potential we calculated the electronic structure of a slab consisting of 51 atomic layers. The resulting one-particle energies and wave functions where employed in the evaluation of the *g* function along the lines described in Ref.^84^. An important novel issue of the present calculations is the inclusion of the electron temperature in the evaluation of the occupation factors $f_{ns\mathbf{k}}$ entering Eq. (9). Since the temperature of the experiment is comparable with the binding energy of the surface state at the bottom, it may have an impact in the surface excitation spectra.

## Data Availability

The data that support the findings of this study are available from the corresponding author upon reasonable request.

## References

[1] Grimvall G (1981). The Electron–Phonon Interaction in Metals.

[2] Weiler M, Dreher L, Heeg C, Huebl H, Gross R, Brandt MS, Goennenwein STB (2011). Elastically driven ferromagnetic resonanse in nickel thin films. Phys. Rev. Lett..

[3] Berk C, Jaris M, Yang W-G, Dhuey S, Cabrini S, Schmidt H (2019). Stronly coupled magnon–phonon dynamics in a single nanomagnet. Nat. Commun..

[4] Kogar A, Rak MS, Vig S, Husain AA, Flicker F, Joe YI, Venema L, MacDougall GJ, Chiang TC, Fradkin E, van Wezel J, Abbamonte P (2017). Signatures of exciton condensation in a transition metal dichalcogenide. Science.

[5] Pines D (1956). Electron interaction in solids. Can. J. Phys..

[6] March NH, Tosi MP (1995). Collective effects in condensed conducting phases including low-dimensional systems. Adv. Phys..

[7] Olego D, Pinczuk A, Gossard AC, Wiegmann W (1982). Plasma dispersion in a layered electron gas: A determination in GaAs-(AlGa)As heterostructures. Phys. Rev. B.

[8] Yu H, Hermanson JC (1989). Collective excitations in the accumulation layer of InAs(110): Nonlocal response theory. Phys. Rev. B.

[9] Diaconescu B, Pohl K, Vattuone L, Savio L, Hofmann P, Silkin VM, Pitarke JM, Chulkov EV, Echenique PM, Farías D, Rocca M (2007). Low-energy acoustic plasmons at metal surfaces. Nature.

[10] Silkin VM, García-Lekue A, Pitarke JM, Chulkov EV, Zaremba E, Echenique PM (2004). Novel low-energy collective excitation at metal surfaces. Europhys. Lett..

[11] Pohl K, Diaconescu B, Vercelli G, Vattuone L, Silkin VM, Chulkov EV, Echenique PM, Rocca M (2010). Acoustic surface plasmon on Cu(111). EPL.

[12] Park SJ, Palmer RE (2010). Acoustic plasmon on the Au(111) surface. Phys. Rev. Lett..

[13] Vattuone L, Smerieri M, Langer T, Tegenkamp C, Pfnür H, Silkin VM, Chulkov EV, Echenique PM, Rocca M (2013). Correlated motion of electrons on the Au(111) surface: Anomalous acoustic surface-plasmon dispersion and single-particle excitations. Phys. Rev. Lett..

[14] Braun K-F, Rieder K-H (2008). Ni(111) surface state observed with scanning tunneling microscopy. Phys. Rev. B.

[15] Lobo-Checa J, Okuda T, Hengsberger M, Patthey L, Greber T, Blaha P, Osterwalder J (2008). Hidden surface states on pristine and H-passivated Ni(111): Angle-resolved photoemission and density-functional calculations. Phys. Rev. B.

[16] Esbjerg N, Nørskov JK (1980). Dependence of the He-scattering potential at surfaces on the surface-electron-density profile. Phys. Rev. Lett..

[17] Senet P, Toennies JP, Benedek G (2002). Theory of the He-phonon forces at metal surfaces. Europhys. Lett..

[18] Sklyadneva IY, Benedek G, Chulkov EV, Echenique PM, Heid R, Bohnen K-P, Toennies JP (2011). Mode-selected electron–phonon coupling in superconducting Pb nanofilms determined from He atom scattering. Phys. Rev. Lett..

[19] Benedek G, Bernasconi M, Bohnen K-P, Campi D, Chulkov EV, Echenique PM, Heid R, Sklyadneva IY, Toennies JP (2014). Unveiling mode-selected electron–phonon interactions in metal films by helium atom scattering. Phys. Chem. Chem. Phys..

[20] Feuerbacher B, Willis RF (1981). Momentum transfer cutoff in the scattering of neon atoms from a nickel (111) surface. Phys. Rev. Lett..

[21] Schönhammer K, Gunnarsson O (1980). Sticking probability on metal surfaces: Contribution from electron–hole–pair excitations. Phys. Rev. B.

[22] Schönhammer K, Gunnarsson O (1980). Localized dynamic perturbation in metals. Z. Phys. B.

[23] Brivio GP, Grimley TB (1979). Non-adiabatic processes in adsorption–desorption phenomena. Surf. Sci..

[24] Gunnarsson O, Schönhammer K (1982). Boson approximations for localized dynamic perturbations in metals. Phys. Rev. B.

[25] Hulpke E, Lüdecke J (1992). Hydrogen-induced phonon anomaly on the W(110) surface. Phys. Rev. Lett..

[26] Hulpke E, Lüdecke J (1993). The giant surface phonon anomaly on hydrogen saturated W(110) and Mo(110). Surf. Sci..

[27] Benedek, G., Pardo, M. & Toennies, J. P. in *Highlights on Spectroscopies of Semiconductors and Nanostructures*, G. Guizzetti, A. C. Andreani, F. Marabelli, and M. Patrini, Eds., Conf. Proc. Vol. 94 (SIF, Bologna, 2007), pp. 151–167.

[28] Benedek G, Toennies J. P. (2018). Atomic Scale Dynamics at Surfaces.

[29] Bortolani V, Franchini A, Nizzoli F, Santoro G, Benedek G, Celli V (1983). Theory of inelastic Ne scattering from the Ni(111) surface. Surf. Sci..

[30] Al Taleb A, Anemone G, Hayes WW, Manson JR, Farías D (2017). Multiphonon excitation and quantum decoherence in neon scattering from solid surfaces. Phys. Rev. B.

[31] Braun J, Toennies JP, Wöll C (1999). Local layer-by-layer growth of Ni on hydrogen-terminated diamond C(111): A combined helium-atom scattering and XPS study. Phys. Rev. B.

[32] Lange G, Toennies JP (1996). Helium-atom-scattering measurements of surface-phonon dispersion curves of the C(111)-H(1x1) surface. Phys. Rev. B.

[33] Hayes, W., Al Taleb, A., Anemone, G., Manson, J. R. & Farías, D. Ne atom scattering from Ir(111) under nearly classical conditions. *Surf. Sci.* **678**, 20–24 (2018).

[34] Feuerbacher, B. Inelastic scattering from metal surfaces. in *Dynamics of Gas-Surface Interaction*, G. Benedek and U. Valbusa, Eds. (Springer, Heidelberg, 1982), pp. 263–272.

[35] Menezes W, Knipp P, Tisdale G, Sibener SJ (1990). Surface phonon spectroscopy of Ni(111) studied by inelastic electron scattering. Phys. Rev. B.

[36] Baroni S, de Gironcoli S, Dal Corso A, Giannozzi P (2001). Phonons and related crystal properties from density-functional perturbation theory. Rev. Mod. Phys..

[37] Velasco VR, Yndurain F (1979). Lattice vibrations at (111) surfaces and stacking faults in transition metals: Ni. Surf. Sci..

[38] Bortolani, V., Franchini, A., Nizzoli, F. & Santoro, G. in *Dynamics of Gas-Surface Interaction*, G. Benedek and U. Valbusa, Eds., (Springer, Heidelberg, 1982), p. 196.

[39] Bortolani V, Franchini A, Nizzoli F, Santoro G (1982). Surface phonon calculations of the Ni(111) surface with adsorbed oxygen. Solid State Commun..

[40] Black JE, Rahman TS, Mills DL (1983). Spectral densities in surface lattice dynamics at large wave vector. Phys. Rev. B.

[41] Black, J. E. in *Dynamical Properties of Solids*, G. K. Horton and A. A. Maradudin, Eds. (Elsevier, Amsterdam, 1990), Chap. 4.

[42] Black JA, Campbell DA, Wallis RF (1981). Dynamical motion of atoms in Ni(001) and Rh(001) surfaces. Surf. Sci..

[43] Black JA, Campbell DA, Wallis RF (1982). Surface vibrations on body centered cubic and face centered cubic metal surfaces: The (100) surfaces. Surf. Sci..

[44] Black JE, Bopp P (1984). The vibration of atoms at high miller index surfaces: Face centred cubic metals. Surf. Sci..

[45] Menezes W, Knipp P, Tisdale G, Sibener SJ (1990). Inelastic electron scattering study of Ni(111) surface phonons. J. Electron Spectr. Rel. Phenom..

[46] Preuss E, Wuttig M, Sheka E, Natkaniec I, Nechitaylov P (1991). The amplitude weighted density of bulk and surface vibrations in a well dispersed nickel. Sov. Phys. JETP.

[47] Ditlevsen PD, Nørskov JK (1991). Vibrational properties of aluminum, nickel and copper surfaces. Surf. Sci..

[48] Büscher H, Klein-Hessling W, Ludwig W (1993). Surface phonons and elastic surface waves. Ann. Phys..

[49] Koleske DD, Sibener SJ (1993). Molecular dynamics simulations of the basal planes of Ni and Cu using Finnis–Sinclair potentials. Surf. Sci..

[50] Benedek G, Bernasconi M, Chis V, Chulkov EV, Echenique PM, Hellsing B, Toennies JP (2010). Theory of surface phonons at metal surfaces: Recent advances. J. Phys. Condens. Matter.

[51] Vidali G, Ihm G, Kim H-Y, Cole MW (1991). Potentials of physical adsorption. Surf. Sci. Rep..

[52] Puska MJ, Nieminen RM, Manninen M (1981). Atoms embedded in an electron gas: Immersion energies. Phys. Rev. B.

[53] Baumberger M, Rieder KH, Stocker W (1986). Analyses of Ne-diffraction data from transition-metal surfaces based on charge-density calculations. Surf. Sci. Lett..

[54] Jackson JM, Mott NF (1932). Energy exchange between inert gas atoms and a solid surface. Proc. Roy. Soc. (Lond.) A.

[55] Liebsch A (1997). Electronic Excitations at Metal Surfaces.

[56] Persson BNJ, Zaremba E (1985). Electron-hole pair production at metal surfaces. Phys. Rev. B.

[57] Pitarke JM, Nazarov VU, Silkin VM, Chulkov EV, Zaremba E, Echenique PM (2004). Theory of acoustic surface plasmons. Phys. Rev. B.

[58] Benedek, G., Manson, J. R., & Miret-Artés, S. The Electron-Phonon Coupling Constant for Single-Layer Graphene on Metal Substrates Determined from He Atom Scattering. *PCCP,*10.1039/d0cp04729 (2020), and arXiv:submit/3453175 [cond-mat.mtrl-sci] 5 Nov 2020. 10.1039/d0cp04729e 33180894

[59] Al Taleb, A., Anemone, G., Miranda, R. & Farías, D. Characterization of interlayer forces in 2D heterostructures using neutral atom scattering. *2D Mater.* **5,** 045002 (2018).

[60] Tamtögl A, Kraus P, Mayrhofer-Reinhartshuber M, Benedek G, Bernasconi M, Dragoni D, Campi D, Ernst WE (2019). Statics and dynamics of multivalley charge density waves in Sb(111). NPJ Quantum Mater..

[61] Ruckhofer A, Campi D, Bremholm M, Hofmann P, Benedek G, Bernasconi M, Ernst WE, Tamtögl A (2020). Terahertz surface modes and electron–phonon coupling in Bi$$_2$$Se$$_3$$(111). Phys. Rev. Res..

[62] McIntosh EM, Kole PR, El-Batanouny M, Chisnall DM, Ellis J, Allison W (2013). Measurement of the phason dispersion of misfit dislocations on the Au(111) surface. Phys. Rev. Lett..

[63] Winter H, Schüller A (2011). Fast atom diffraction during grazing scattering from surfaces. Progr. Surf. Sci..

[64] Bundaleski N, Khemliche H, Soulisse P, Roncin P (2008). Grazing incidence diffraction of keV Helium atoms on a Ag(110) surface. Phys. Rev. Lett..

[65] Barredo D, Laurent G, Nieto P, Farías D, Miranda R (2010). High-resolution elastic and rotationally inelastic diffraction of D-2 from NiAl (110). J. Chem. Phys..

[66] Al Taleb, A., Anemone, G., Farías, D. & Miranda, R. Acoustic surface phonons of graphene on Ni(111). *Carbon* **99**, 416–422 (2016).

[67] Giannozzi, P., Baroni, S., Bonini, N., Calandra, M., Car. R., Cavazzoni, C., Ceresoli, D., Chiarotti, G. L., Cococcioni, M., Dabo, I., *et al.* QUANTUM ESPRESSO: A modular and open-source software project for quantum simulations of materials. *J. Phys. Condens. Matter* **21**, 395502 (2009). 10.1088/0953-8984/21/39/395502 21832390

[68] Vanderbilt D (1990). Soft self-consistent pseudopotentials in a generalized eigenvalue formalism. Phys. Rev. B.

[69] Perdew JP, Burke K, Ernzerhof M (1996). Generalized gradient approximation made simple. Phys. Rev. Lett..

[70] Monkhorst HJ, Pack JD (1976). Special points for Brillouin-zone integrations. Phys. Rev. B.

[71] Runge E, Gross EKU (1984). Density-functional theory for time-dependent systems. Phys. Rev. Lett..

[72] Petersilka M, Gossmann UJ, Gross EKU (1996). Excitation energies from time-dependent density-functional theory. Phys. Rev. Lett..

[73] Kresse G, Furthmüller J (1996). Efficiency of ab-initio total energy calculations for metals and semiconductors using a plane-wave basis set. Comput. Mater. Sci..

[74] Kresse G, Furthmüller J (1996). Efficient iterative schemes for ab initio total-energy calculations using a plane-wave basis set. Phys. Rev. B.

[75] http://www.vasp.at

[76] Silkin VM, Chulkov EV, Echenique PM (2003). First-principles calculation of the electron inelastic mean free path in Be metal. Phys. Rev. B.

[77] Silkin VM, Chulkov EV, Echenique PM (2004). Band structure versus dynamical exchange-correlation effects in surface plasmon energy and damping: A first-principles calculation. Phys. Rev. Lett..

[78] Chulkov EV, Silkin VM, Echenique PM (1999). Image potential states on metal surfaces: Binding energies and wave functions. Surf. Sci..

[79] Magaud L, Pasturel A, Mallet P, Pons S, Veuillen J-Y (2004). Spin-polarized Shockley state at Ni(111) free surface and at Ni-Cu-based structures on Cu(111) surface. Europhys. Lett..

[80] Ohwaki T, Wortmann D, Ishida H, Blügel S, Terakura K (2006). Phys. Rev. B.

[81] Dzemiantsova LV, Karolak M, Lofink F, Kubetzka A, Sachs B, von Bergmann K, Hankemeier S, Wehling TO, Frömter R, Oepen HP, Lichtenstein AI, Wiesendanger R (2011). Multiscale magnetic study of Ni(111) and graphene on Ni(111). Phys. Rev. B.

[82] Donath M, Passek F, Dose V (1993). Surface state contribution to the magnetic moment of Ni(111). Phys. Rev. Lett..

[83] Okuda T, Lobo-Checa J, Auwärter W, Morscher M, Hoesch M, Petrov VN, Hengsberger M, Tamai A, Dolocan A, Cirelli C, Corso M, Muntwiler M, Klöckner M, Roos M, Osterwalder J, Greber T (2009). Exchange splitting of the three $$\overline{\Gamma }$$ surface states of Ni(111) from three-dimensional spin- and angle-resolved photoemission spectroscopy. Phys. Rev. B.

[84] Silkin VM, Pitarke JM, Chulkov EV, Echenique PM (2005). Acoustic surface plasmons in the noble metals Cu, Ag, and Au. Phys. Rev. B.

